# Polymorphism of IL‐12/IL‐23 axis is associated with coronary heart disease

**DOI:** 10.1111/jcmm.18100

**Published:** 2024-01-08

**Authors:** Jiangtao Dong, Qianwen Chen, Tian Xie, Mengru Wang, Mengqi Wang, Lingfeng Zha

**Affiliations:** ^1^ Department of Cardiology, Union Hospital, Tongji Medical College Huazhong University of Science and Technology Wuhan China; ^2^ Hubei Key Laboratory of Biological Targeted Therapy, Union Hospital, Tongji Medical College Huazhong University of Science and Technology Wuhan China; ^3^ Hubei Provincial Engineering Research Center of Immunological Diagnosis and Therapy for Cardiovascular Diseases, Union Hospital, Tongji Medical College Huazhong University of Science and Technology Wuhan China; ^4^ Department of Cardiovascular Surgery Union Hospital, Tongji Medical College, Huazhong University of Science and Technology Wuhan China; ^5^ Maternal and Child Health Hospital of Hubei Province, Tongji Medical College Huazhong University of Science and Technology Wuhan China; ^6^ Key Laboratory of Molecular Biophysics of Ministry of Education, College of Life Science and Technology, Center for Human Genome Research Cardio‐X Institute, Huazhong University of Science and Technology Wuhan China

**Keywords:** coronary heart disease, genetic, *IL12B*, polymorphism, rs2853694

## Abstract

*IL12B* encodes the shared p40 subunit (IL‐12p40) of IL‐12 and IL‐23, which have diverse immune functions and are closely related to the occurrence and development of atherosclerosis (AS). However, the exact role of *IL12B* in coronary heart disease (CHD) was still unknown. A case–control association analysis was performed between five single nucleotide polymorphisms (SNPs) of *IL12B* (rs1003199, rs3212219, rs2569254, rs2853694 and rs3212227) and CHD in Chinese Han population (1824 patients with CHD vs. 2784 controls). Logistic regression analyses were used to study the relationships between SNPs and CHD, while multiple linear regression analyses were used to test the association between the SNP and the severity of CHD. In addition, the plasma IL12B concentration of CHD patients were detected by ELISA. We detected a significant association between one of the SNPs, rs2853694^−G^ and CHD (*p*_adj_ = 2.075 × 10^−5^, OR, 0.773 [95% CI, 0.686–0.870]). Stratified analysis showed that rs2853694 was associated with CHD in both male and female populations and was significantly associated with both early‐ and late‐onset CHD. In addition, rs2853694 is also related to the different types of CHD including clinical‐CHD and anatomical‐CHD. Moreover, there are significant differences in serum IL12B concentrations between rs2853694^−TT^ carriers and rs2853694^−GT^ carriers in CHD patients (*p* = 0.010). A common variant of *IL12B* was found significantly associated with CHD and its subgroups. As a shared subunit of IL‐12 and IL‐23, IL‐12p40 may play a key role in IL‐12/IL‐23 axis mediated AS, which is expected to be an effective therapeutic target for CHD.

## 1 INTRODUCTION

Coronary heart disease (CHD) is an ischemic heart disease in which blood vessels narrow due to coronary atherosclerosis (AS), resulting in myocardial infarction (MI), hypoxia and even necrosis. Coronary heart disease, as a high incidence of cardiovascular diseases (CVDs), is one of the highest mortality diseases globally. It affects as many as 11 million people in China, and the annual growth rate of 20%, there are 1 million deaths due to CHD every year. Coronary heart disease has caused serious economic burden and health burden to the world, so it is urgent to seek effective prevention and treatment strategies of CHD.

In recent years, accumulating studies indicated inflammation are closely related to the progression of CHD.^1^, ^2^ Interleukins (ILs) are inflammatory cytokines involved in pro‐inflammatory and anti‐inflammatory responses through interactions with a variety of receptors, such as Toll‐like receptors (TLRs).^3^ Research has suggested that ILs and TLRs could represent promising targets for the therapeutic intervention of inflammation‐associated diseases.^4^ The pathogenic role of inflammation is well‐established, and many animal studies have suggested that anti‐inflammatory therapy can inhibit the progression of AS.^5^ The CANTOS study found that the use of the IL‐1β blocking antibody (canakinumab) significantly reduced cardiovascular events in patients with CHD. It confirmed that IL‐1β is an effective target for anti‐inflammatory therapy in CHD in the complex inflammatory regulatory network.^6^, ^7^ Based on JUPITER studies, some scholars have proposed that the therapeutic effect of statins on CHD is partly due to the anti‐inflammatory effect.^8^ However, due to the complex regulatory network of inflammatory response, how to find out the key regulatory factors in this network and use them as targets to effectively regulate inflammation is the key to the success of anti‐inflammatory therapy.

Results from epidemiological surveys, twin studies and relative risk studies all show a significant genetic predisposition to CHD.^9^, ^10^, ^11^ Relatives of people with CHD have a 2 to 3.9 times increased risk of developing CHD compared with relatives without CHD. Although numerous susceptibility genes for CHD have been discovered through genetic studies, these susceptibility genes can only explain about 28% of the heritability of CHD, and these conclusions are mainly drawn from western European populations.^12^ Moreover, many of those susceptibility genes for CHD have not yet been elucidated. The full genetic basis of CHD and its molecular mechanism of action in the disease through various research strategies is still needed, so as to provide theoretical basis for precision medicine in the future.

**FIGURE 1:**
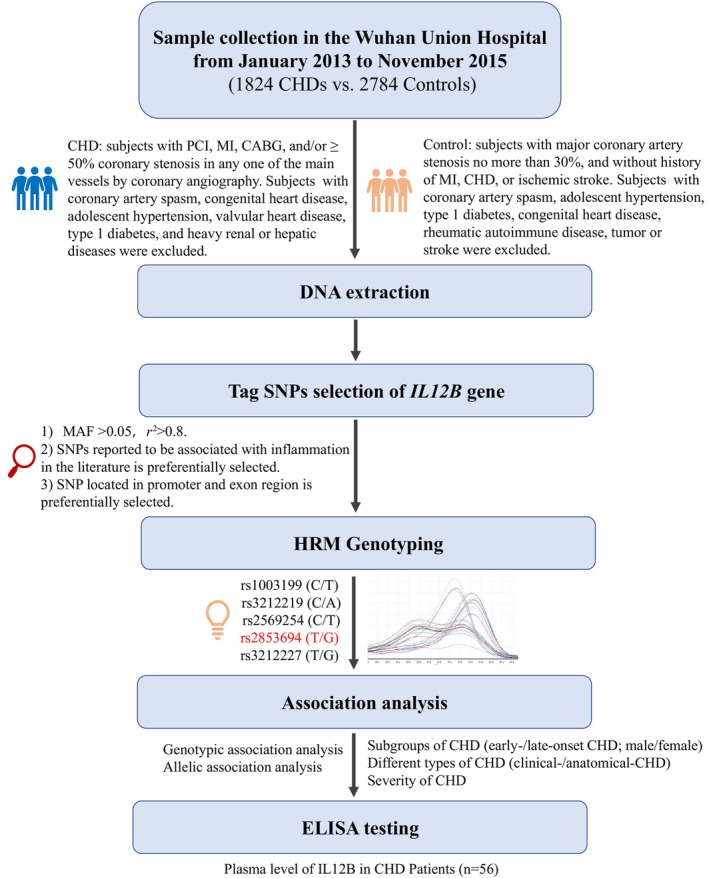
Study design flow chart.

*IL12B* encodes the p40 subunit (IL‐12p40) of IL‐12 and IL‐23, which have been reported to be closely related to AS. The expressions of both IL‐12 and IL‐23 are increased in human atherosclerotic plaques, and IL‐12 promoted the formation of AS in mice.^13^, ^14^ Currently, IL‐12/IL‐23 axis is considered a therapeutic target for autoimmune inflammation,^15^ which may also be a new target for treating AS.^16^ Studies have found that polymorphism on *IL12B* is associated with susceptibility to some inflammatory diseases,^17^, ^18^, ^19^ and in some disease states, the polymorphism on *Il12B* regulate IL‐12p40 and IL‐23 transcription and affects their serum levels.^20^, ^21^ In addition, recent research has found *IL12B* polymorphisms are associated with the presence of premature CHD and with cardiovascular risk factors in Mexican population.^22^ However, genetic relationship between *IL12B* and CHD in the Chinese population is unclear. Our study explores the role of *IL12B* in the genetic factors of CHD by studying the association between its polymorphisms and CHD (Figure 1), in order to find the susceptible polymorphism of CHD, and further understand the role of IL‐12/IL‐23 axis in the occurrence and development of CHD, so as to clarify the pathological process of CHD.

## 2 MATERIALS AND METHODS

### 2.1 Study objects

**FIGURE 2:**
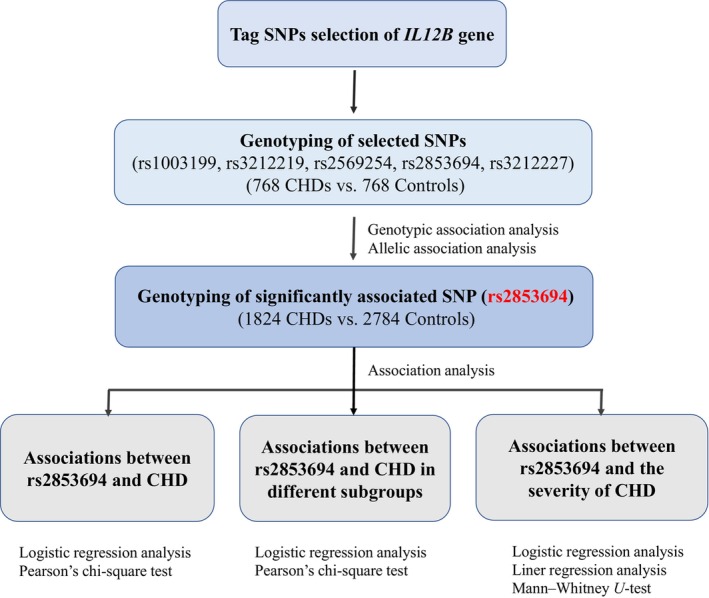
Flow chart of the association analysis between *IL12B* and CHD.

This study collected a total of 1824 patients with CHD and 2784 controls in the Wuhan Union Hospital (Wuhan, China) from January 2013 and November 2015. Of these, 768 CHDs and 768 controls were in the Phase I discovery study, and the remaining samples were used for further extended sample validation of single nucleotide polymorphism (SNP) that showed significant result in Phase I study (Figure 2). All of our samples have been obtained with DNA and relevant clinical information including: age, gender, height, weight, the history of smoking, drinking, hypertension and diabetes, blood lipid and fasting blood glucose, as well as other biochemical detection indicators information.^23^ In addition, there are ECG, CT, heart colour ultrasound, coronary angiography and other imaging data of patients with CHD. The inclusion and exclusion criteria for CHD and control were as described in our previous public article^24^, ^25^ (Figure 1). According to the different diagnostic criteria, we divided the CHD patients into two subgroups. The anatomical‐CHD group was defined as severe coronary stenosis (≥50% coronary stenosis in any one of the main vessels [right coronary artery, left anterior descending, left main and left circumflex artery]) which was diagnosed by coronary angiography and the clinical‐CHD group is MI or revascularization which is by clinical diagnosis. The severity of CHD was assessed using Gensini score performed according to coronary angiography. The coronary segment's score was judged as 32, 16, 8, 4, 2 and 1 for its stenosis of 100%, 99–91%, 90–76%, 75–51%, 50–26% and 25–0%, respectively. And the coronary segment's score was multiplied with a specific factor judged as the vessel importance and size (ranging from 5.0 to 0.5) and the total sum weights of the segments is the Gensini index for each patient.^26^ Our study meets the requirements of the World Medical Association Declaration of Helsinki and has been approved by the local ethics committee.

### 2.2 Tag SNPs selection of *IL12B*

**FIGURE 3:**
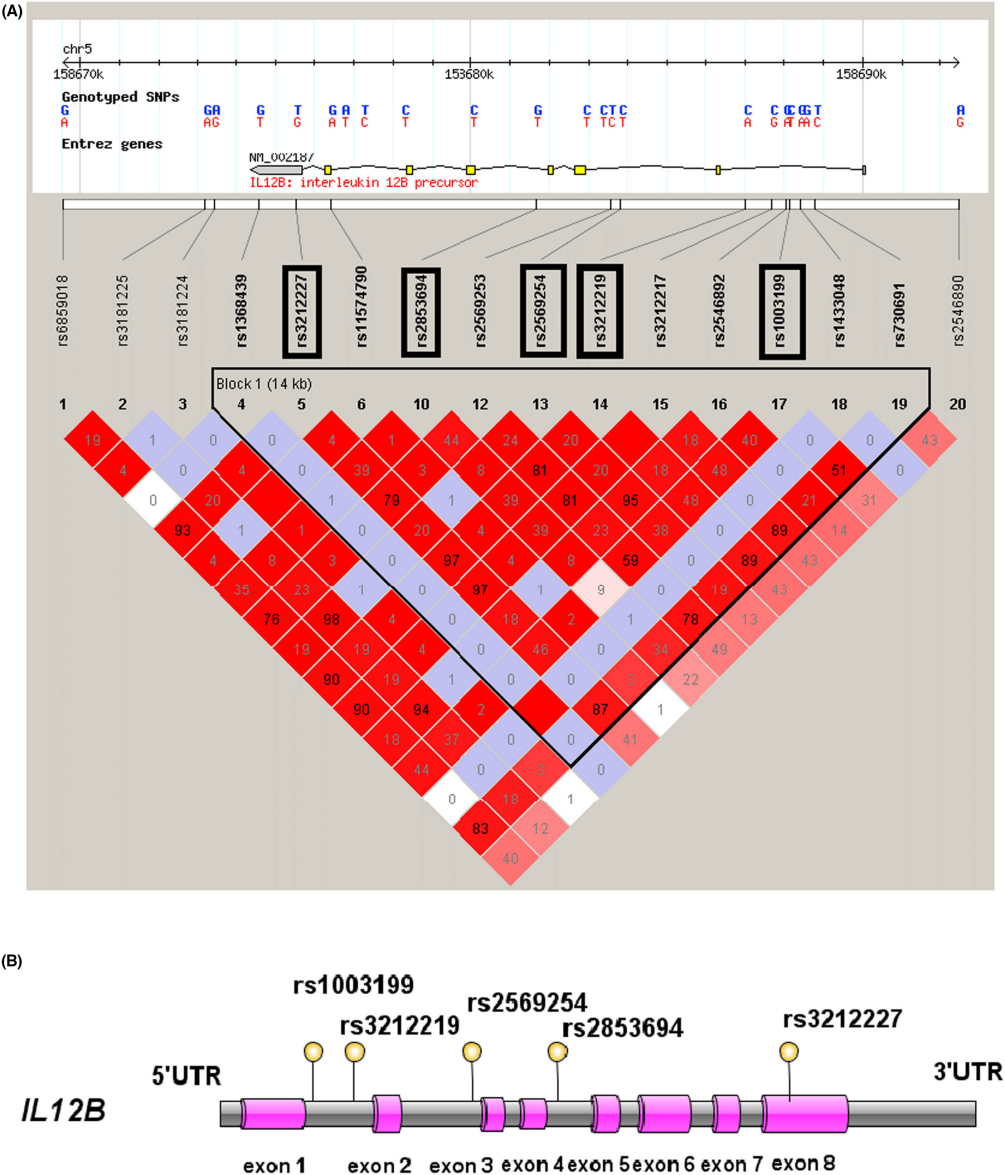
(A) Haploview linkage disequilibrium (LD) block of *IL12B* region based on the HapMap CHB and JPT data Sets. Each diamond represents the LD degree between the SNPs. The number within each diamond represents the *r* ^2^ value. The colour indicates the D′: regions of high LD are showed in dark red; regions of low LD are showed in white. The selected Tag SNPs are showed in black box. (B) The location of the selected SNPs on the *IL12B* region.

A Linkage disequilibrium (LD) Block of CHB + JPT (China and Japan) population was established in the Haploview software (V4.2 version) covering the entire *IL12B* gene and its upstream and downstream 5 kbp region (Figure 3). The Tag SNPs selection principles were as follows^24^: (1) Minimum allele frequency (MAF) >0.05, *r* ^2^ > 0.8. (2) SNPs reported to be associated with inflammation in the literature is preferentially selected. (3) SNP located in promoter and exon region is preferentially selected. According to the selection principle above, a total of five SNPs were included in our study, namely rs1003199, rs3212219, rs2569254, rs2853694 and rs3212227 (Figure 2). For detailed SNP information, see Table **S1**.

### 2.3 PCR amplification and high‐resolution melt (HRM) genotyping

DNA was amplified in a 25 μL volume of PCR system with 2.5 μL 10× PCR buffer, 0.5 μL dNTP, 0.5 μL forward/reverse primer for each SNP (Table S2), 1.5 μL DNA, 0.5 μL Taq DNA polymerase, 0.5 μL fluorochrome (SYTO 9) and 18.5 μL double steaming water with ABI 9700 PCR Amplifier (Applied Biosystem, USA).^24^ The PCR products were genotyped by Rotor‐Gene Q High Resolution Melt system (QIAGEN, Germany).^27^

### 2.4 Association analysis

In this study, age, body mass index (BMI), total cholesterol (Tch), triglyceride (TG), high‐density lipoprotein cholesterol (HDL‐C) and low‐density lipoprotein cholesterol (LDL‐C) levels were tested by independent sample t‐test to test whether there were differences in the mean values of these clinical indicators between the two groups and their respective populations. Traditional risk factors for CHD were corrected as covariables using logistic regression analysis, and multiple linear regression was carried out to analysis the effects of rs2853694 on Gensini scores (SPSS, v.16.0). Allelic and genotypic association analysis were carried out by 2 × 2 and/or 2 × 3 pearson chi‐squared contingence tables respectively.^27^ Mann–Whitney *U*‐tests evaluated the relationship between different genotypes of rs2853694 and LN of Gensini scores.^24^ As five SNPs were covered in this association analysis, the statistically significant *p* value was ≤0.01 (0.05/5) after Bonferroni correction.

### 2.5 Plasma level of IL12B in CHD patients

A total of 56 CHD patients were selected from the combined population for analysis of the association between rs2853694 genotypes and the plasma level of IL12B. Blood samples were collected in tubes containing EDTA as an anticoagulant. After centrifuging blood samples for 15 min at 1000 *g* at 4°C within 30 min of collection, supernatant was obtained. 100 μL plasma of each sample was aspirated for ELISA test. According to the manufacturer's protocol (R&D Systems), standard recombined human IL12B provided by this kit was diluted to eight different concentrations to generate standard curves. After incubation, colour development was measured at 420 nm by means of a spectrophotometer and OD value was gained for each sample. Based on the standard curve drew by the different concentrations of standard recombined human IL12B, we got the corresponding IL12B concentration of each sample. The correlations among the rs2853694 genotypes and IL12B plasma concentrations were studied by non‐parametric test.

## 3 RESULTS

### 3.1 Characteristics of study objects

In the Phase I discovery study, we compared the characteristics of those groups (768 CHDs vs. 768 controls) and found that age and BMI of the CHD group were higher than those of the control group, and the proportion of males, smoking, hypertension and diabetes in the CHD group were also higher compared with the control group. In addition, the Tch, TG and LDL‐C concentrations were significantly higher compared with the control group, while the HDL‐C concentration was lower compared with the control group. For the subsequent study, 1824 CHDs and 2784 controls were included, and the characteristics of those samples were similar to those of phase I. See Table 1 for details.

**TABLE 1:** Characteristics of the study population.

Characteristics	Discovery‐population		*p*	Combined‐population		*p*
	CHD (*n* = 768)	Control (*n* = 768)		CHD (*n* = 1824)	Control (*n* = 2784)	
Age (years)	63.33 ± 11.05	61.05 ± 10.52	3.682 × 10^−5^	62.41 ± 11.24	54.69 ± 12.58	<10^−6^
Male (%)	71.48	50.52	<10^−6^	72.75	58.58	<10^−6^
Smoking (%)	46.48	27.73	<10^−6^	45.39	17.06	<10^−6^
BMI (kg/m^2^)	24.24 ± 1.54	23.68 ± 1.38	<10^−6^	24.32 ± 1.58	23.69 ± 1.41	<10^−6^
Hypertension (%)	68.62	52.34	<10^−6^	67.65	22.49	<10^−6^
DM (%)	34.77	15.76	<10^−6^	32.18	6.57	<10^−6^
Tch (mmol/L)	5.09 ± 1.18	4.88 ± 1.24	0.001	5.13 ± 1.17	4.83 ± 1.05	<10^−6^
TG (mmol/L)	1.79 ± 1.16	1.48 ± 0.95	1.479 × 10^−3^	1.81 ± 1.25	1.50 ± 0.99	<10^−6^
HDL‐C (mmol/L)	1.12 ± 0.30	1.26 ± 0.32	<10^−6^	1.11 ± 0.29	1.32 ± 0.31	<10^−6^
LDL‐C (mmol/L)	2.98 ± 1.02	2.74 ± 0.91	<10^−6^	3.03 ± 1.05	2.72 ± 0.84	<10^−6^

*Note*: The data are shown as the mean ± SD.

Abbreviations: BMI, body mass index; CHD, coronary heart disease; DM, diabetes mellitus; HDL‐C, high‐density lipoprotein cholesterol; LDL‐C, low‐density lipoprotein cholesterol; Tch, total cholesterol; TG, triglyceride.

### 3.2 Allelic and genotypic association analysis of SNPs in *IL12B* gene with CHD in discovery population

In the phase I discovery study, only rs2853694 of the five SNPs in the *IL12B* gene was significantly associated with CHD by allele association analysis before correcting for traditional risk factors (*p*_obs_ = 2.790 × 10^−5^). The other four SNPs (rs1003199, rs3212219, rs2569254 and rs3212227) were not associated with CHD (all *p*_obs_ > 0.05). When correcting the traditional risk factor, rs2853694^−G^ remained significantly associated with CHD (*p*_adj_ = 3.904 × 10^−5^, OR, 0.694 [95% CI, 0.583–0.826]). The other four SNPs remained unassociated with CHD after correction (all *p*_adj_ > 0.05) (Table 2).

**TABLE 2:** Allelic association analysis between *IL12B* and CHD in discovery population.

Population	SNP‐allele	*N*		MAF		*P*obs	*p*_adj_	OR (95% CI)
		Case (*n* = 768)	Control (*n* = 768)	Case	Control			
Discovery	rs1003199^T^	728	701	0.327	0.328	0.281	0.426	1.075 (0.899–1.286)
	rs3212219^A^	748	661	0.438	0.411	0.147	0.068	1.171 (0.989–1.386)
	rs2569254^T^	672	712	0.163	0.163	0.999	0.990	0.998 (0.794–1.255)
	rs2853694^G^	750	737	0.297	0.369	2.790 × 10^−5^	3.904 × 10^−5^	0.694 (0.583–0.826)
	rs3212227^G^	672	678	0.455	0.433	0.256	0.098	1.157 (0.974–1.375)

Abbreviations: MAF, minimum allele frequency; OR (95% CI), odds ratio (95% confidence interval) after adjustment; *p*_adj_, *p* value adjusted by the covariates (age, gender, BMI, hypertension, diabetes mellitus, smoking history, Tch, TG, HDL‐C and LDL‐C); *p*_obs_, observed *p* value.

In genotypic association analysis, rs1003199 was related to CHD in ADD model before correcting for risk factors (*p*_obs_ = 0.046), rs2853694 was significantly associated with CHD in all three genotype modes (*p*_obs_ = 8.358 × 10^−5^ for ADD model; *p*_obs_ = 2.305 × 10^−4^ for DOM model; *p*_obs_ = 0.001 for REC model). However, after adjusting for risk factors, rs1003199 was no longer associated with CHD (*p*_adj_ = 0.354), while the correlation between rs2853694 and CHD still exists (*p*_adj_ = 2.192 × 10^−5^ for ADD model; *p*_adj_ = 1.561 × 10^−4^ for DOM model; *p*_adj_ = 0.005 for REC model). In addition, after correcting for risk factors, rs3212227 was associated with CHD in a DOM model (*p*_adj_ = 0.046), but did not pass multiple correction tests (Table 3).

**TABLE 3:** Genotypic association analysis between *IL12B* and CHD in discovery population.

Population	SNP‐allele	Model	*N*		*p*_obs_	*p*_adj_	OR (95% CI)
			Case (*n* = 768)	Control (*n* = 768)			
Discovery	rs1003199^T^	ADD	29/418/281	37/358/306	0.046	0.354	1.103 (0.896–1.359)
		DOM	447/281	395/306	0.052	0.085	1.235 (0.972–1.569)
		REC	29/699	37/664	0.244	0.115	0.627 (0.351–1.120)
	rs3212219^A^	ADD	160/335/253	130/283/248	0.339	0.081	1.154 (0.982–1.356)
		DOM	495/253	413/248	0.148	0.076	1.251 (0.977–1.602)
		REC	160/588	130/531	0.425	0.287	1.172 (0.875–1.571)
	rs2569254^T^	ADD	19/181/472	20/192/500	1.000	0.990	0.998 (0.795–1.253)
		DOM	200/472	212/500	0.996	0.994	1.001 (0.770–1.301)
		REC	19/653	20/692	0.984	0.951	0.978 (0.478–1.999)
	rs2853694^G^	ADD	55/335/360	91/362/284	8.358 × 10^−5^	2.192 × 10^−5^	0.674 (0.562–0.809)
		DOM	390/360	453/284	2.305 × 10^−4^	1.561 × 10^−4^	0.630 (0.498–0.797)
		REC	55/695	91/646	0.001	0.005	0.564 (0.379–0.841)
	rs3212227^G^	ADD	123/365/184	121/345/212	0.282	0.087	1.169 (0.978–1.398)
		DOM	488/184	466/212	0.117	0.046	1.313 (1.005–1.714)
		REC	123/549	121/557	0.827	0.509	1.112 (0.811–1.525)

Abbreviations: ADD, additive mode, rs1003199_TT/CT/CC; rs3212219_AA/CA/CC; rs2569254_TT/CT/CC; rs2853694_GG/GT/TT; rs3212227_GG/GT/TT; DOM, dominant mode, rs1003199_TT + CT/CC; rs3212219_AA+CA/CC; rs2569254_TT + CT/CC; rs2853694_GG + GT/TT; rs3212227_GG + GT/TT; REC, recessive model, rs1003199_TT/CT + CC; rs3212219_AA/CA + CC; rs2569254_TT/CT + CC; rs2853694_GG/GT + TT; rs3212227_GG/GT + TT; OR (95% CI), odds ratio (95% confidence interval) after adjustment; *p*_adj_, *p* value adjusted by the covariates (age, gender, BMI, hypertension, diabetes mellitus, smoking history, Tch, TG, HDL‐C and LDL‐C); *p*_obs_, observed *p* value.

### 3.3 Allelic and genotypic association analysis of rs2853694 in *IL12B* gene with CHD in combined population

Through the analysis of relatively small samples in the phase I discovery stage, we found that a SNP in *IL12B* gene, rs2853694, was significantly correlated with CHD. Therefore, we expanded the sample size to further explore the relationship between rs2853694 and CHD. Finally, we found for allele analysis, rs2853694^−G^ were significantly associated with CHD in more than 4500 samples (1824 CHDs vs. 2784 controls) (*p*_obs_ = 0.004), remained significant after adjusting for risk factors (*p*_adj_ = 2.075 × 10^−5^, OR, 0.773 [95% CI, 0.686–0.870]). In genotype analysis, rs2853694 was significantly associated with CHD in the ADD (GG/GT/TT) and REC (GG/GT + TT) models (*p*_obs_ = 0.008 for ADD model; *p*_obs_ = 0.007 for REC model), the DOM (GG + GT/TT) model is also associated with CHD (*p*_obs_ = 0.024). The association between rs2853694 and CHD remained after correction for risk factors (*p*_adj_ = 1.424 × 10^−5^ for ADD model; *p*_adj_ = 3.981 × 10^−5^ for DOM model; *p*_adj_ = 0.008 for REC model) (Table 4).

**TABLE 4:** Allelic and genotypic association analysis between rs2853694 and CHD in combined population.

Population	SNP‐allele	Model	*N*		*p*_obs_	*p*_adj_	OR (95% CI)
			Case (*n* = 1787)	Control (*n* = 2572)			
Combination	rs2853694^G^	ALLE	1123/2451	1770/3374	0.004	2.075 × 10^−5^	0.773 (0.686–0.870)
		DOM	964/823	1476/1096	0.024	3.981 × 10^−5^	0.717 (0.611–0.840)
		REC	159/1628	294/2278	0.007	0.008	0.700 (0.537–0.913)
		ADD	159/805/823	294/1182/1096	0.008	1.424 × 10^−5^	0.763 (0.676–0.862)

Abbreviations: ADD, additive mode, rs2853694_GG/GT/TT; DOM, dominant mode, rs2853694_GG + GT/TT; OR (95% CI), odds ratio (95% confidence interval) after adjustment; *p*_adj_, *p* value adjusted by the covariates (age, gender, BMI, hypertension, diabetes mellitus, smoking history, Tch, TG, HDL‐C and LDL‐C); *p*_obs_, observed *p* value; REC, recessive model, rs2853694_GG/GT + TT.

### 3.4 Association of rs2853694 in *IL12B* gene with CHD in subgroup populations

Considering the influence of gender and age on CHD, we conducted different stratified analyses according to gender and age of onset of CHD. First of all, we divided the population of CHD into early‐onset CHD and late‐onset CHD based on the age of onset (early‐onset CHD: the first onset age ≤55 years for male and ≤65 years for female; late‐onset CHD: the first onset age >55 years for male and >65 years for female). Allele analysis showed that rs2853694^−G^ was significantly associated with both types of CHD (*p*_adj_ = 3.549 × 10^−4^ for early‐onset CHD; *p*_adj_ = 0.009 for late‐onset CHD). Genotype analysis showed that rs2853694 was associated with early‐onset CHD in ADD (GG/GT/TT) and DOM (GG + GT/TT) models (*p*_adj_ = 2.716 × 10^−4^ for ADD model; *p*_adj_ = 7.135 × 10^−5^ for DOM model), rs2853694 was associated with late‐onset CHD in the ADD (GG/GT/TT) and REC (GG/GT + TT) modes (*p*_adj_ = 0.007 for ADD model; *p*_adj_ = 0.005 for REC model) (Table 5).

**TABLE 5:** Association analysis between rs2853694 and CHD in the onset age subgroups.

Population	SNP allele	Model	CHD early‐onset (626 cases vs. 2572 controls)		CHD late‐onset (1161 cases vs. 2572 controls)	
			*p*_adj_	OR (95% CI)	*p*_adj_	OR (95% CI)
Combination	rs2853694^G^	ALLE	3.549 × 10^−4^	0.739 (0.626–0.872)	0.009	0.820 (0.707–0.951)
		ADD	2.716 × 10^−4^	0.728 (0.614–0.864)	0.007	0.812 (0.698–0.946)
		DOM	7.135 × 10^−5^	0.642 (0.516–0.799)	0.070	0.831 (0.679–1.015)
		REC	0.179	0.775 (0.533–1.125)	0.005	0.627 (0.451–0.871)

*Note*: The early‐onset CHD group contained subjects with onset age of CHD ≤65 years for females and ≤55 years for males; the late‐onset CHD group contained subjects with onset age of CHD > 65 years for females and >55 years for males.

Abbreviations: ADD, additive mode, rs2853694_GG/GT/TT; DOM, dominant mode, rs2853694_GG + GT/TT; OR (95% CI), odds ratio (95% confidence interval) after adjustment; *p*_adj_, *p* value adjusted by the covariates (age, gender, BMI, hypertension, diabetes mellitus, smoking history, Tch, TG, HDL‐C and LDL‐C); REC, recessive model, rs2853694_GG/GT + TT.

Second, we divided the population into male and female according to sex, and the results showed that in allele analysis, rs2853694^−G^ was significantly correlated with CHD in both male and female groups (*p*_adj_ = 0.004 for male; *p*_adj_ = 4.311 × 10^−4^ for female). Further genotype analysis showed that rs2853694 was also correlated with CHD in ADD (GG/GT/TT) and DOM (GG + GT/TT) models (*p*_adj_ = 0.003 for ADD model and *p*_adj_ = 0.010 for DOM model in male group; *p*_adj_ = 3.691 × 10^−4^ for ADD model and *p*_adj_ = 1.793 × 10^−4^ for DOM model in female group) (Table 6).

**TABLE 6:** Allelic and genotypic association analysis of rs2853694 with CHD in gender subgroups.

Population	SNP‐allele	Model	Male (1301 cases vs. 1513 controls)		Female (486 cases vs. 1059 controls)	
			*p*_adj_	OR (95% CI)	*p*_adj_	OR (95% CI)
Combination	rs2853694^G^	ALLE	0.004	0.805 (0.696–0.932)	4.311 × 10^−4^	0.690 (0.561–0.848)
		DOM	0.010	0.771 (0.634–0.939)	1.793 × 10^−4^	0.592 (0.451–0.779)
		REC	0.029	0.697 (0.504–0.965)	0.118	0.690 (0.433–1.099)
		ADD	0.003	0.796 (0.685–0.925)	3.691 × 10^−4^	0.680 (0.550–0.841)

Abbreviations: ADD, additive mode, rs2853694_GG/GT/TT; DOM, dominant mode, rs2853694_GG + GT/TT; OR (95% CI), odds ratio (95% confidence interval) after adjustment; *p*_adj_, *p* value adjusted by the covariates (age, BMI, hypertension, diabetes mellitus, smoking history, Tch, TG, HDL‐C and LDL‐C); REC, recessive model, rs2853694_GG/GT + TT.

### 3.5 Associations between rs2853694 in *IL12B* and different types of CHD

Coronary heart disease has different clinical manifestations due to different pathological anatomy and pathophysiology. To explore the influence of rs2853694 on different types of CHD, we divided CHD population into clinical‐CHD and anatomical‐CHD. The anatomical‐CHD group was defined as severe coronary stenosis and the clinical‐CHD group is MI or revascularization. Our results found that in allele analysis, rs2853694^−G^ was associated with both types of CHD (*p*_adj_ = 2.750 × 10^−5^ for clinical‐CHD; *p*_adj_ = 0.001 for anatomical‐CHD). Genotype analysis showed that rs2853694 was also associated with two types of CHD under three genotype patterns (*p*_adj_ = 2.013 × 10^−5^ for ADD model; *p*_adj_ = 3.384 × 10^−5^ for DOM model; *p*_adj_ = 0.016 for REC model in clinical‐CHD; *p*_adj_ = 0.001 for ADD model; *p*_adj_ = 0.001 for DOM model; *p*_adj_ = 0.041 for REC model in anatomical‐CHD) (Table 7).

**TABLE 7:** Association analysis of rs2853694 with CHD in the disease status subgroups.

Population	SNP‐allele	Model	Clinical‐CHD (1546 cases vs. 2572 controls)		Anatomical‐CHD (1289 cases vs. 2572 controls)	
			*p*_adj_	OR (95% CI)	*p*_adj_	OR (95% CI)
Combination	rs2853694^G^	ALLE	2.750 × 10^−5^	0.766 (0.677–0.868)	0.001	0.803 (0.705–0.914)
		ADD	2.013 × 10^−5^	0.758 (0.667–0.861)	0.001	0.795 (0.696–0.908)
		DOM	3.384 × 10^−5^	0.703 (0.596–0.831)	0.001	0.752 (0.632–0.895)
		REC	0.016	0.711 (0.539–0.937)	0.041	0.741 (0.556–0.987)

*Note*: The anatomical‐CHD group was defined as severe coronary stenosis and the clinical‐CHD group is MI or revascularization.

Abbreviations: ADD, additive mode, rs2853694_GG/GT/TT; DOM, dominant mode, rs2853694_GG + GT/TT; OR (95% CI), odds ratio (95% confidence interval) after adjustment; *p*_adj_, *p* value adjusted by the covariates (age, gender, BMI, hypertension, diabetes mellitus, smoking history, Tch, TG, HDL‐C and LDL‐C); REC, recessive model, rs2853694_GG/GT + TT.

### 3.6 Associations between rs2853694 in *IL12B* and the severity of CHD

It is also possible that rs2853694 is associated with the severity of CHD. We evaluated the severity of CHD using Gensini scoring system for each patient with CHD. Since Gensini score data in the sample did not conform to normal distribution, logarithmic conversion (LN) of Gensini score data was performed before analysis. First, we use linear regression model to conduct quantitative trait association. Our analysis results showed that rs2853694 was not related to the severity of CHD either in allele mode or genotype mode (*p* > 0.05) (Table 8). Then we divide the LN of Gensini scores into quartiles, 4th (highest) and 1st (lowest) quartiles of the LN of Gensini scores were choose to conduct case–control association analysis. Results showed that rs2853694 was not related to the severity of CHD either in allele mode or genotype mode (*p* > 0.05) (Table 8). In addition, we evaluated the relationship between different genotypes of rs2853694 and LN of Gensini scores through Mann–Whitney U‐tests. Results also showed no difference between LN of Gensini scores and rs2853694 genotypes (*p* > 0.05) (Figure **S1**). These results suggest that there is no correlation between rs2853694 and the degree of CHD.

**TABLE 8:** Association analysis of rs2853694 with the LN of Gensini scores.

SNP‐allele	Model	Quantitative trait association (*n* = 1375)				Case–control association (*n* = 1375)			
		Beta	SE	*r* ^2^	*p*_adj_	*N*		*p*_adj_	OR (95% CI)
						1st	4th		
rs2853694^G^	ALLE	−0.008	0.033	0.045	0.659	744	688	0.395	0.903 (0.714–1.143)
	ADD	−0.012	0.034	0.041	0.652	372	344	0.374	0.894 (0.699–1.144)
	DOM	−0.006	0.043	0.041	0.813	372	344	0.571	0.914 (0.670–1.247)
	REC	−0.016	0.075	0.041	0.549	372	344	0.311	0.743 (0.419–1.319)

*Note*: The 1st quartile was defined as the quartile with the lowest LN of Gensini scores, and the 4th quartile was defined as the quartile with the highest LN of Gensini scores.

Abbreviations: ADD, additive mode, rs2853694_GG/GT/TT; DOM, dominant mode, rs2853694_GG + GT/TT; OR (95% CI), odds ratio (95% confidence interval) after adjustment; *p*_adj_, *p* value adjusted by the covariates (age, gender, BMI, hypertension, diabetes mellitus, smoking history, Tch, TG, HDL‐C and LDL‐C); REC, recessive model, rs2853694_GG/GT + TT.

### 3.7 Association of rs2853694 genotypes with plasma IL12B concentration in CHD patient

Among the 56 CHD patients, the detection rate of plasma IL12B concentrations was 100% (median, 194.81 pg/mL; range, 35.68–505.07 pg/mL).

**FIGURE 4:**
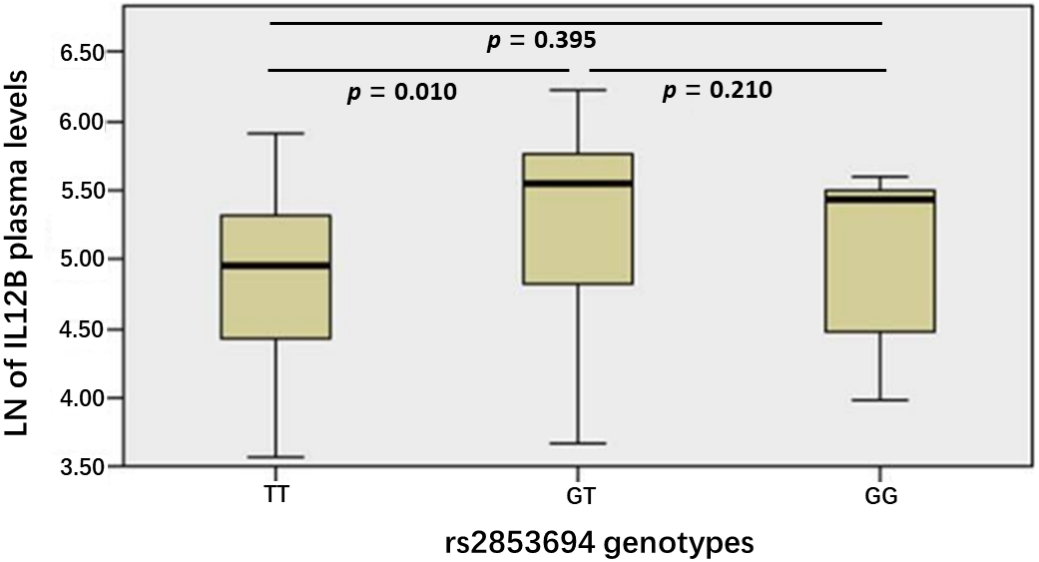
Plasma levels of IL12B according to rs2853694 genotype.

There were 7 rs2853694^−GG^ carriers (median, 176.19 pg/mL; range, 53.31–271.85 pg/mL), 25 rs2853694^−GT^ carriers (median, 239.61 pg/mL; range, 39.21–505.07 pg/mL), 24 rs2853694^−GG^ carriers (median, 153.56 pg/mL; range, 35.68–372.23 pg/mL). There were significant differences in plasma IL12B concentration between rs2853694^−TT^ carriers and rs2853694^−GT^ carriers (*p* = 0.010) (Figure 4).

## 4 DISCUSSION

Our study examined the relationship between SNPs of *IL12B* and CHD in a Chinese Han population. We detected a significant association between one of the SNPs, rs2853694 and CHD in a small sample. Further significant correlation between rs2853694 and CHD remained in the expanded sample. Stratified analysis showed that rs2853694 was associated with CHD in both male and female populations and was significantly associated with both early‐ and late‐onset CHD. In addition, rs2853694 is also related to the different types of CHD including clinical‐CHD and anatomical‐CHD. However, no association was found between rs2853694 and the severity of CHD. In addition, there are significant differences in serum IL12B concentrations between rs2853694^−TT^ carriers and rs2853694^−GT^ carriers in CHD patients.

*IL12B* gene located on chromosome 5q31, a region where we have previously identified multiple CHD susceptibility SNPs.^23^, ^26^, ^28^ *IL12B* belongs to the IL‐12 family, consisting of IL‐12, IL‐23, IL‐27 and IL‐35. Our previous research found that other genes in the IL‐12 family are related to CHD.^25^, ^29^ 9p21.3 is the first CHD susceptibility site identified by genome‐wide association studies (GWASs). Zollbrecht et al.^30^ found 9p21.3 haplotype can affect the expression of *IL12B*, which is higher up‐regulated in 9p21.3 risk haplotype carrier. Recently, researcher^22^ found rs1363670 of *IL12B* was associated with a low risk of premature CHD (pCHD) in Mexican population and in pCAD patients, the rs2853694 of *IL12B* was related to lower risk of insulin resistance. However, Morahan et al.^31^ found rs2853694 was not susceptible to type 1 diabetes. Moreover, *IL12B* or IL‐12 were not related to insulin sensitivity.^32^ In addition, Chung et al.^33^ found rs2853694 was associated with coronary AS in patients with rheumatoid arthritis (RA) with a relatively small sample (140 RA patients for Caucasian). In our study, we found rs2853694 was associated with CHD including both early‐ and late‐onset CHD, moreover, our results also found that rs2853694 were associated with different disease states of CHD. Another SNP of *IL12B*, rs3212227 was found to be no associated with MI in British Caucasian population^34^ and not related to both presence or severity of CHD in Japanese population.^35^ Similarly, no correlation was found between rs3212227 and CHD in our study.

In addition, our results found that there were significant differences in plasma IL12B concentration between rs2853694^−TT^ carriers and rs2853694^−GT^ carriers of CHD patient although rs2853694 is located in the intron region. As we all know, most of the disease susceptibility SNPs found in GWAS are located in the non‐coding regions of genes, and the specific mechanisms of most of the SNPs have not been clarified due to the limitations of research methods. However, with the continuous efforts of scientists, many valuable databases have been established to help predict the possible mechanisms of genetic variation in non‐coding regions.^36^ Through various prediction websites, such as HaploReg database, RegulomeDB, ENCODE, we found that the region where rs2853694 located contained a large number of regulatory elements, such as enhancer markers H3K27ac, H3K4me1, H3K27me3 and transcription factor binding sites. We speculated that although rs2853694 is located in the intron region, this region may be an enhancer region. When rs2853694^−T^ change to rs2853694^−G^, it may initiate some transcriptional regulation by binding different transcriptions or changing the way of binding to the transcriptions, affecting the expression of IL12B. However, the specific regulatory mechanism is worthy of further investigation in our subsequent experiments.

*IL12B* encodes the p40 subunit (IL‐12p40) of IL‐12 and IL‐23, which closely related to the progression of AS. Plasma IL‐12 level are obviously elevated in AS or CAD,^37^, ^38^, ^39^ which also elevated in APOE^−/−^ mice, and elevated IL‐12 are related to the progression of AS.^40^ Recombinant IL‐12 administered to APOE^−/−^ and LDLR^−/−^ mice can increase atherosclerotic plaque areas and exacerbate AS, while blocking IL‐12 significantly reduced this effect.^41^, ^42^ All of these studies suggest that IL‐12 have pro‐atherogenic effects, indicated that it may be a prospective therapeutic target. Although there is a lack of evidence that IL‐23 acts directly on AS, increasing studies show that IL‐23 also has the effect of promoting AS. For CHD patients undergoing percutaneous coronary intervention drug‐eluting stents, peripheral blood mononuclear cell circulating IL‐23 concentration were higher in patients with in‐stent restenosis.^43^ Subramanian et al.^44^ found granulocyte‐macrophage colony stimulating factor increases the expression of IL‐23, which further promotes the differentiation of macrophages and the development of AS.

Although both IL‐12 and IL‐23 belong to the IL‐12 family of cytokines, they have different immune functions, particularly in determining the nature of the immune response to be undertaken. IL‐12 is a key factor promoting Th1 differentiation,^45^ while IL‐23 is the key factor promoting Th17 differentiation.^46^ Th1 and Th17 cell‐mediated immune inflammation paly key role in the occurrence and development of AS.^47^, ^48^, ^49^ In clinical studies, our previous research found that the proportion of Th17 cells, Th17 characteristic transcription factor RORγt and Th17‐related cytokines (IL‐17, IL‐23, IL‐6) in peripheral blood of patients with acute coronary syndrome (ACS) of CHD were significantly increased, suggesting that Th17 cells mediated the instability of AS plaques.^50^ Th1/Th2 dysfunction exists in AS animals and patients with CHD, and inhibition of Th1 function can delay the progression of AS.

IL‐12 and IL‐23 share the IL‐12p40 subunit. Similarly, IL‐12 and IL‐23 receptors share the IL‐12Rβ1 subunit. Therefore, previous interventions targeting IL‐12 are likely to actually act on IL‐23 by acting on IL‐12p40 or IL‐12Rβ1 subunits, that is, interventions targeting Th1 actually act on Th17 cells. This made scholars puzzled about the pathogenesis of diseases mediated by Th1 cells. RNA levels of IL‐12p40 in asymptomatic carotid plaques were significantly higher than the symptomatic samples.^51^ Another study found compared to APOE^−/−^ mice, IL‐12p40^−/−^APOE^−/−^ mice showed reduction of lesion because of reduced aggregation of macrophages in the aortic root.^52^ Recently study found a significant difference between the percentage methylation of promoter DNA of *IL12B* in CHD patients and control subjects.^53^ The author holds that maybe the M1/M2 macrophage polarization marker. The balance of M1/M2 macrophages is closely related to the occurrence and development. Zhou et al.^54^ suggest that curcumin can be a therapeutic for AS, as curcumin can inhibit M1 macrophage polarization and the production of TNF‐α, IL‐6, and IL‐12p40. Nikolajuk et al.^32^ found IL‐12p40 was positive associated with fat mass and TG, while negative associated with HDL‐C in obese women. As a shared subunit of IL‐12 and IL‐23, IL‐12p40 may pivotal in IL‐12 and IL‐23 mediated AS. Currently, there are monoclonal antibodies targeting IL‐12/IL‐23 axis, which have shown good efficacy in the treatment of other inflammatory diseases by blocking the IL‐12/IL‐23 pathway.^55^ Considering the important role of IL‐12p40 in IL‐12/IL‐23 axis, it is expected to be a key target for the treatment of AS.

This study is the first to investigate the relationship between *IL12B* gene and CHD from a genetic perspective in a Chinese Han population. The results showed that a SNP of *IL12B* is related to CHD, which provides a new target for targeted therapy of CHD and provides a theoretical basis for individualized therapy. But limitations remained in this subject: people in different regions may have some differences in genetic background and disease susceptibility due to the inconsistency of geographical environment and living environment; the majority of the samples in this study are from the Han population in central China, and the samples of people in northern China or other areas can be collected for verification in subsequent studies; lacking the laboratory data regarding the inflammatory state of the patients and the sample size for detecting IL12B concentration was small. In addition, this study only found the susceptibility sites and possible pathogenesis of CHD from the perspective of genetics through statistical methods. Further functional experiments are needed to verify the effects of rs2853694 on IL‐12p40 and the IL‐12/IL‐23 axis.

## 5 CONCLUSION

Our study investigated the relationship between *IL12B* gene and CHD from a genetic perspective in a Chinese Han population. A common variant of *IL12B* was found significantly associated with CHD and its subgroups. As a shared subunit of IL‐12 and IL‐23, IL‐12p40 may play a key role in IL‐12/IL‐23 axis mediated AS, which is expected to be an effective therapeutic target for CHD.

## AUTHOR CONTRIBUTIONS

**Jiangtao Dong:** Validation (equal); visualization (equal). **Qianwen Chen:** Formal analysis (equal); methodology (equal). **Tian Xie:** Investigation (equal); resources (equal). **Mengru Wang:** Software (equal). **Mengqi Wang:** Data curation (equal). **Lingfeng Zha:** Conceptualization (equal); writing – review and editing (supporting).

## FUNDING INFORMATION

Our work was funded by the National Natural Science Foundation of China of Lingfeng Zha (No. 82200319), Hubei Provincial Natural Science Foundation of China of Tian Xie (2023AFB1101), Health Commission of Hubei Province of China (WJ2023M033).

## CONFLICT OF INTEREST STATEMENT

The authors declare no conflicts of interest.

## Supporting information


Appendix S1.

## Data Availability

All data in the article can be obtained by contacting the corresponding author of the article.
