# Supplementary material for: Polymorphism of IL‐12/IL‐23 axis is associated with coronary heart disease
Source: J Cell Mol Med. 2024 Jan 8;28(3):e18100. doi: 10.1111/jcmm.18100 (PMC10844691; doi:10.1111/jcmm.18100)
Supplement: Supplementary file 1 — Appendix S1. [file JCMM-28-e18100-s001.docx]

Supplementary Material

# Supplementary Tables and Figures

**Table S1.** **Information for selected Tag SNPs of *IL12B***

| **Gene** | **SNP** | **Variant** | **MAF** | **Alle** |
| --- | --- | --- | --- | --- |
| ***IL12B*** | rs1003199 | Intron variant | 0.37 | C/T |
|  | rs3212219 | Intron variant | 0.36 | C/A |
|  | rs2569254 | Intron variant | 0.12 | C/T |
|  | rs2853694 | Intron variant | 0.36 | T/G |
|  | rs3212227 | 3 prime UTR variant | 0.36 | T/G |

**Table S2. Primer sequences for PCR**

| **Gene** | **SNP** | **Forward primer (**5'-3') | **Reverse primer (**5'-3') | **HRM temperature** |
| --- | --- | --- | --- | --- |
| ***IL12B*** | rs1003199 | GTGCACATTTCCCTACTGCC | AGAGGAAGAGGTAAGGGAACA | 74­°C-78­°C |
|  | rs3212219 | AGAAGCTAAACCCCTACAGCA | TTGGGTTAGGGACAGGAACG | 80­°C-85­°C |
|  | rs2569254 | TGCAAAGCCCAAGAGACAAA | GGCCAACGATCTAAGCATGG | 78­°C-83­°C |
|  | rs2853694 | AAATTGCCTGGCCATGCTTC | CCCTAAGATCTACGCCCTGG | 79­°C-83­°C |
|  | rs3212227 | CAACGGAATAGACCCAAAAAGATA | GGCAACTTGAGAGCTGGAAAATC | 75­°C-79­°C |


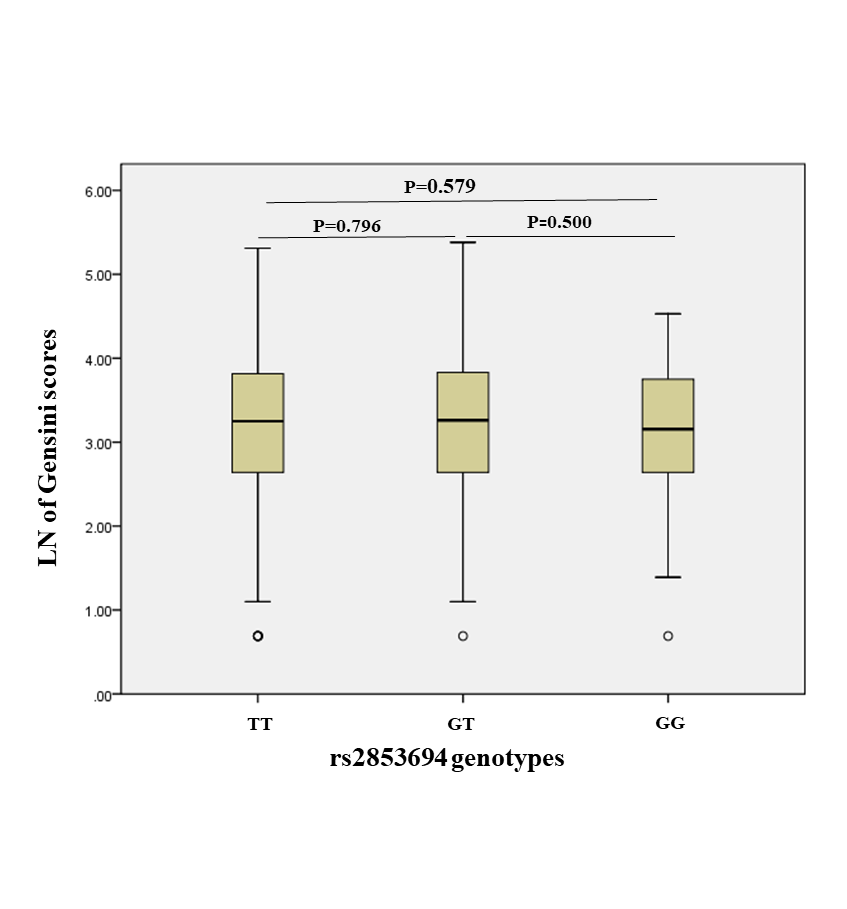


**Figure S1.** **Association analysis between the LN of Gensini scores and the genotypes of rs2853694**

The distribution difference of the LN of the Gensini scores in different genotypes of rs2853694 was compared by Mann-Whitney U-test.
